# Thin Films Based on Cobalt Phthalocyanine:C60 Fullerene:ZnO Hybrid Nanocomposite Obtained by Laser Evaporation

**DOI:** 10.3390/nano10030468

**Published:** 2020-03-05

**Authors:** Marcela Socol, Nicoleta Preda, Andreea Costas, Bogdana Borca, Gianina Popescu-Pelin, Andreea Mihailescu, Gabriel Socol, Anca Stanculescu

**Affiliations:** 1 National Institute of Materials Physics, 077125 Magurele, Romania; andreea.costas@infim.ro (A.C.); bogdana.borca@infim.ro (B.B.); sanca@infim.ro (A.S.); 2 National Institute for Lasers, Plasma and Radiation Physics, 077125 Magurele, Romania; gianina.popescu@inflpr.ro (G.P.-P.); andreea.mihailescu@inflpr.ro (A.M.); gabriel.socol@inflpr.ro (G.S.)

**Keywords:** cobalt phthalocyanine, ZnO nanoparticles, hybrid nanocomposite films, MAPLE, photovoltaic effect

## Abstract

Matrix-assisted pulsed laser evaporation (MAPLE) was used to deposit hybrid nanocomposite thin films based on cobalt phthalocyanine (CoPc), C60 fullerene and ZnO nanoparticles. The inorganic nanoparticles, with a size of about 20 nm, having the structural and optical properties characteristic of ZnO, were chemically synthesized by a simple precipitation method. Furthermore, ZnO nanoparticles were dispersed in a dimethyl sulfoxide solution in which CoPc and C60 had been dissolved, ready for the freezing MAPLE target. The effect of the concentration of ZnO nanoparticles on the structural, morphological, optical and electrical properties of the CoPc:C60:ZnO hybrid nanocomposite layers deposited by MAPLE was evaluated. The infrared spectra of the hybrid nanocomposite films confirm that the CoPc and C60 preserve their chemical structure during the laser deposition process. The CoPc optical signature is recognized in the ultraviolet–visible (UV–Vis) spectra of the obtained layers, these being dominated by the absorption bands associated to this organic compound while the ZnO optical fingerprint is identified in the photoluminescence spectra of the prepared layers, these disclosing the emission bands linked to this inorganic semiconductor. The hybrid nanocomposite layers exhibit globular morphology, which is typical for the thin films deposited by MAPLE. Current-voltage (J-V) characteristics of the structures developed on CoPc:C60:ZnO layers reveal that the addition of an appropriate amount of ZnO nanoparticles in the CoPc:C60 mixture leads to a more efficient charge transfer between the organic and inorganic components. Due to their photovoltaic effect, structures featuring such hybrid nanocomposite thin films deposited by MAPLE can have potential applications in the field of photovoltaic devices.

## 1. Introduction

Hybrid nanocomposites, materials that combine the advantages of both organic and inorganic components, have currently received increased interest due to their potential applications in a large variety of technological areas such as optics, electronics, energy, medicine [1,2,3,4]. In the field of electronics, the organics are particularly appealing due to the possibility of tuning their properties through the corresponding molecular design according to required functionalities [4] while the inorganic materials are attractive especially for their stability and high mobility [5]. Additionally, the organic compounds are highly flexible and compatible with plastic substrates, being the most suitable candidates for wearable electronics [4].

In the field of solar cells, even the structures containing organic materials (OSC) as active layer present lower electrical parameters in comparison with those based on inorganic compounds, their efficiency has recently exceeded 17% [6]. The OSC limitations related to the low mobility of the organic semiconductors or to an inefficient charge transport can be overcome by adding inorganic semiconductors in order to obtain hybrid structures [5,7]. Thus, the organic and inorganic components can be used as separate layers or mixed into a bulk heterojunction (BHJ) [5].

The metallic phthalocyanines are small molecule compounds frequently used in the solar cells because of their interesting absorption properties and augmented chemical and thermal stability [8,9]. From the phthalocyanines group, cobalt phthalocyanine (CoPc) is characterized by a good electrical conductivity, being successfully applied in electrocatalysis, organic based field-effect transistors, gas sensors and memory devices [10]. In the OSC structures, the metallic phthalocyanines are good electron donor candidates that can be combined with C60 fullerene which acts as acceptor material and assures the exciton dissociation interface [9].

Metal oxides are known to be one of the most promising inorganic semiconductors that can be incorporated as nanoparticles in BHJ [7]. From all metal oxides, ZnO is of maximum interest because it can be synthesized in structures with different morphologies (wires, prisms, rods, platelets, etc.) through safe, inexpensive, simple wet and dry preparation methods [11,12,13], and all these architectures finding applications in various areas, e.g., water splitting [14], photocatalysis [15], field effect transistors [13], surfaces with special wettability [11]. Among the wet chemical methods, the precipitation reaction represents a straightforward and high throughput path for obtaining ZnO nanoparticles based on the facts that the technique does not require complex apparatus and involves available, cheap raw reagents which favor the fabrication of nanomaterials in large amounts.

The selection of the most appropriate approach for fabricating the hybrid organic-inorganic structures depends on the device architecture (stacked layers or with BHJ). However, in the case of BHJ, spin-coating is the most applied preparation method, other techniques such as doctor blading, ink-jet printing or matrix assisted pulsed laser evaporation (MAPLE) [16,17] have recently started to be used. Laser evaporation-based approach developed to deposit organic thin films with either preservation or minimal degradation of the chemical structure of the raw materials, MAPLE involves a solid target obtained by freezing a mixture containing the organic material dissolved in a compatible solvent. Up to now, various materials like biomaterials, polymers, small molecule organic semiconductors or inorganic nanoparticles were deposited by MAPLE for different applications ranging from the medical area (drug-delivery systems, implant coatings, etc.) to electronics field (organic or hybrid solar cells, sensors, field-effect transistors, etc.) [18]. The advantages such as the deposition of uniform coatings from a small quantity of materials (the concentration of the materials in the solid target is usually lower than 5%) and the fabrication of complex layer structures (the deposited films are very thin with a controllable thickness) make this laser-assisted coating deposition technique suitable for the growth of hybrid thin films. The most important advantage of MAPLE is probably the potential to tune the properties of the hybrid organic-inorganic nanocomposite thin films by engineering the target composition, in this way controlling the performance of the device fabricated with these layers. Furthermore, MAPLE can be considered as a feasible alternative for the deposition of hybrid thin films onto flexible, thermally sensitive or tri-dimensional substrates, and these peculiarities emphasize the technological potential of the MAPLE in the development of photovoltaic devices. Thus, conducting polymer-fullerene derivate [19], metal phthalocyanines [20] or perovskites [21] thin films have been recently obtained by MAPLE for OSC applications.

Therefore, the aim of this study is to deposit the hybrid nanocomposite thin films based on cobalt phthalocyanine (CoPc), C60 fullerene and ZnO nanoparticles by MAPLE. The prepared organic-inorganic layers were thoroughly characterized for emphasizing the influence of the nanoparticles amount on their morphological, structural, compositional, optical and electrical properties. The structures developed with CoPc:C60:ZnO layers present photovoltaic effect confirming their potential application in the field of photovoltaic devices.

## 2. Experimental

All chemical compounds (zinc acetate, sodium hydroxide, cobalt phthalocyanine and C60 fullerene) and the solvent (dimethyl sulfoxide) were purchased from Sigma-Aldrich (Saint Louis, MO, USA) and used without further purification.

ZnO nanoparticles were chemically synthesized by modifying a precipitation procedure described in reference [22]. Thus, under vigorous stirring, a 0.25 M NaOH aqueous solution being added drop wise to a 0.1 M Zn(CH_3_COO)_2_ aqueous solution. After 1 h at 70 °C, the obtained white powder was collected through centrifugation, washed several times with demineralized water and absolute ethanol and dried at room temperature.

Indium tin oxide (ITO)/glass purchased from Ossila, glass and infrared transparent double side polished (100) silicon slides were used as substrates in the MAPLE deposition. Dimethyl sulfoxide (DMSO) was chosen as a solvent in the MAPLE process due to its good absorption at the used laser wavelength and to the solubility of the organic materials (CoPc and C60) in it. The inorganic compound (ZnO nanoparticles) was dispersed in various amounts in the organic mixture. The thin films were deposited at room temperature using a KrF* excimer laser source (Coherent, CompexPro 205, λ = 248 nm, τ_FWHM_ −25 ns) and the following experimental conditions: 240 mJ/cm^2^ laser fluence, 6 cm distance between target and substrate, 65,000 laser pulses, 20 Hz repetition rate, 4 × 10^−4^ mBar pressure in the process chamber. Regardless the substrate type (glass, silicon or ITO/glass), these MAPLE experimental parameters were used in the deposition of all investigated thin films. More details about this laser deposition technique are given in the references [19,23].

**Scheme 1:**
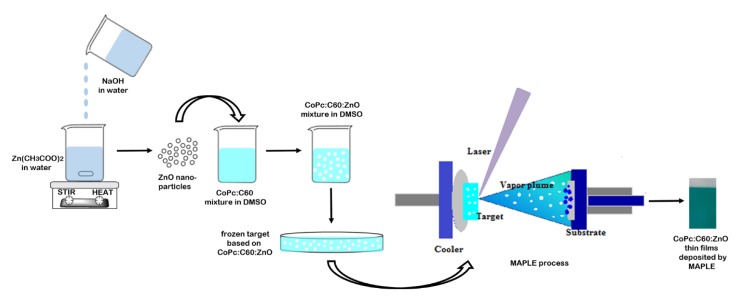
Schematic illustration of the steps involved in the fabrication of CoPc:C60:ZnO hybrid nanocomposite layers deposited by matrix-assisted pulsed laser evaporation (MAPLE).

In order to evidence the influence of inorganic nanoparticles amount on the performance of the hybrid nanocomposite thin films, the concentration of CoPc and C60 in the DMSO was maintained constant at 3% w/v while the weight concentration of ZnO was varied in respect to that of C60, both materials acting as n-type compounds in our structures. For comparison reasons, films containing only the organic mixture (CoPc and C60) or the inorganic material (ZnO nanoparticles) were also deposited. Thus, depending on the inorganic compound concentration in the CoPc:C60:ZnO hybrid composite layers, the investigated samples were labeled as follows: P0 (1:1:0), P1 (1:1:0.25), P2 (1:1:0.75) and P3 (1:1:1). A schematic representation of the stages involved in the chemical synthesis of the ZnO nanoparticles, their integration into the CoPc:C60 mixture, and finally the deposition of hybrid nanocomposite thin films by MAPLE is depicted in Scheme 1.

Furthermore, the chemically synthesized ZnO powder and the MAPLE-deposited thin films were characterized using complementary techniques: Fourier transformed infrared spectroscopy (FTIR), X-ray diffraction (XRD), field-emission scanning electron microscopy (FESEM), energy-dispersive X-ray analysis (EDX), atomic force microscopy (AFM), reflectance spectroscopy (R), ultraviolet–visible spectroscopy (UV–Vis), photoluminescence (PL) and current-voltage measurements (J-V).

Thus, the thickness of the prepared films was estimated using an Ambios Technology XP 100 profilometer (Santa Cruz, CA, USA), the value being in fact the average media of the measurements carried out in three different points. The structure was investigated by a Bruker D8 Advance set-up in a Bragg-Bretano geometry with a Cu K_α1_ (λ = 1.4506 Å) monochromatized radiation. The vibrational characteristics were analyzed with a Shimadzu 800 infrared spectrometer. The morphology and the elemental composition were studied using a Zeiss Merlin Compact field emission scanning electron microscope and a Zeiss EVO 50XVP scanning electron microscope (Oberkochen, Germany) equipped with an energy dispersive X-ray analysis QUANTAX Bruker 200 accessory (Billerica, MA, USA) while the surface roughness parameters were evaluated using a Nanonics Multiview 4000 atomic force microscope. The optical properties were investigated at room temperature involving a Perkin Elmer Lambda 45 UV-Vis spectrophotometer (Waltham, MA, USA) equipped with an integrating sphere, a Carry 5000 spectrophotometer and a FL 920 Edinburgh Instruments spectrometer with a 450 W Xe lamp excitation and double monochromators on both excitation and emission, respectively. The UV-Vis and PL investigations were performed on films deposited on glass and silicon substrates, respectively. For the electrical measurements, aluminum (Al) was selected as back contact. This metal is frequently used in organic and hybrid structures due to its low work function [5,24]. Thus, 110 nm of Al were deposited on all investigated samples, through a shadow mask, by thermal vacuum evaporation employing a Tectra system (2 × 10^−6^ mBar, 300 W, Frankfurt, Germany). The current-voltage measurements were performed at room temperature, under illumination with a LOT-Oriel solar simulator (AM 1.5) using a Keithley 2602B System Source Meter (Cleveland, OH, USA).

## 3. Results and Discussion

**Figure 1:**
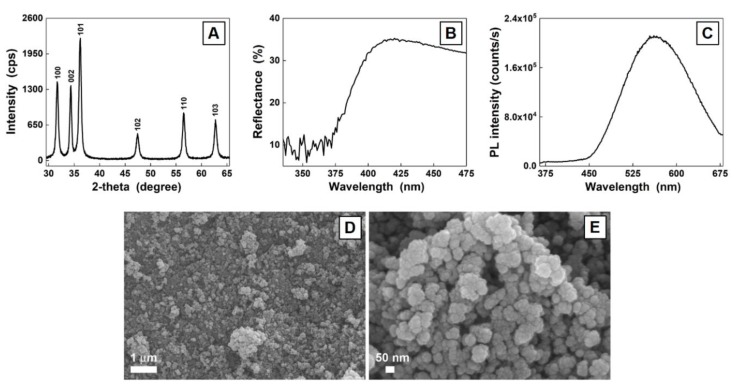
X-ray diffraction (XRD) pattern (**A**), reflectance (R) spectrum (**B**), photoluminescence (PL) spectrum (**C**) and field-emission scanning electron microscopy (FESEM) images (**D**,**E**) of the ZnO nanoparticles synthesized by precipitation.

The structural, optical and morphological properties of the chemically synthesized ZnO white powder are presented in Figure 1. Thus, the main peaks from 2θ: 31.67°, 34.34°, 36.20°, 47.44°, 56.48° and 62.72° in the XRD pattern (Figure 1A) correspond to the Miller indexes of the reflecting planes for (100), (002), (101), (102), (110) and (103), respectively assigned to the hexagonal wurtzite phase of ZnO (JCPDS file no. 36-1451). The strong and sharp diffraction peaks confirm that the as-obtained compound is well crystallized.

The reflectance spectrum (Figure 1B) displays a strong decrease at ~400 nm linked to the ZnO band-to-band transition while the photoluminescence spectrum (Figure 1C) reveals only a strong and broad emission band centered at ~560 nm, this emission being usually related to point defects such as: oxygen vacancy, zinc vacancy, interstitial oxygen, interstitial zinc, hydroxyl group, etc. [11,13,25,26,27]. Typically, ZnO evidences two emission bands: one in the UV domain due to the band-edge emission and another in the visible region linked to the defect emission [25]. In our case, an explanation for the unclear identification of the UV emission takes into account a quenching effect induced by the high density of point defects present in the ZnO synthesized by wet chemical approaches [11,13,27]. From morphological point of view, FESEM images (Figure 1D,E) show that the synthesized powder contains quasi-monodispersed aggregates (Figure 1D) formed by nanoparticles having sizes of about 20 nm (Figure 1E).

**Figure 2:**
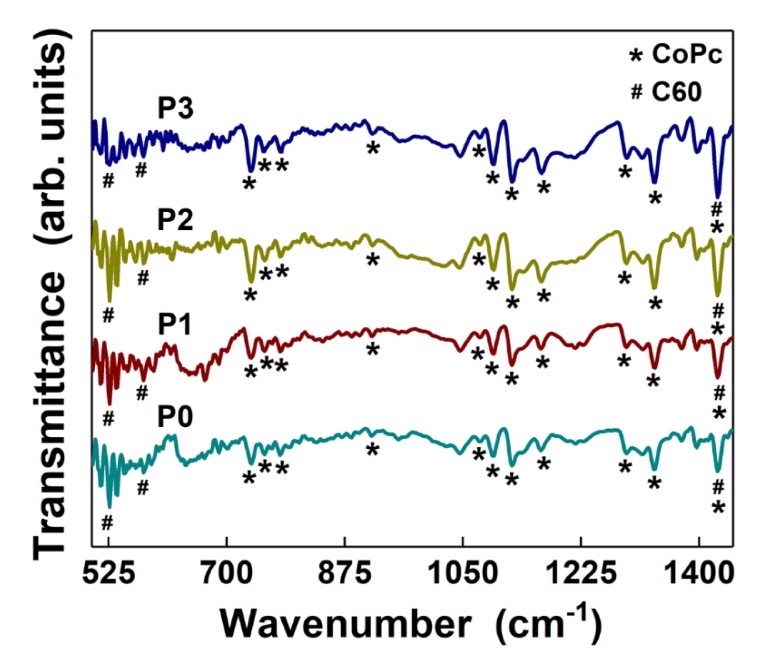
Fourier transformed infrared spectroscopy (FTIR) spectra of the MAPLE-deposited thin films.

The preservation/degradation of the chemical structure of both organic components (CoPc and C60) during the laser deposition process was evaluated from the FTIR spectra of the thin films prepared by MAPLE (Figure 2).

Thus, all FTIR spectra disclose the CoPc β-form absorption bands at about: (i) 736 cm^−1^ due to the C–H out of plane deformation; (ii) 756 cm^−1^ attributed to Co–N bond vibration; (iii) 781 cm^−1^ related to the C=N in plane stretching; (iv) 913 cm^−1^ linked to the metal ligand vibrations; (v) 1075 cm^−1^ and 1090 cm^−1^ due to the C–H in plane deformation; vi) 1122 cm^−1^ and 1166 cm^−1^ related to the C–H in plane bending; (vii) 1290 cm^−1^, 1333 cm^−1^ and 1427 cm^−1^ linked to the C–N and C–C stretching in isoindole [28]. Also, the FTIR spectra reveals absorption bands at 525 cm^−1^, 575 cm^−1^, and 1427 cm^−1^ which are considered the vibrational signature of C60 fullerene [29]. It has to be noticed that the absorption band from 1427 cm^−1^ can be associated with both organic compounds. Consequently, the presence of the characteristic absorption bands assigned to CoPc and C60 in the investigated FTIR spectra confirm that the organic components maintain their chemical structure (no chemical decomposition effect occurs) during the MAPLE deposition.

**Figure 3:**
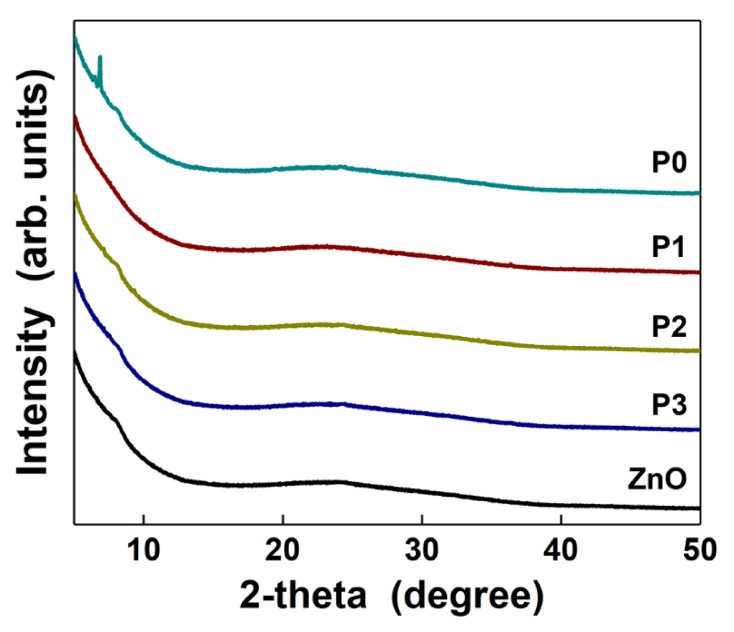
XRD patterns of the MAPLE-deposited thin films.

The structure of the thin films deposited by MAPLE was analyzed from their XRD patterns (Figure 3), an amorphous feature being observed in the case of all hybrid nanocomposite thin films. Nevertheless, for the sample containing only the CoPc:C60 mixture, the characteristic diffraction line, located at ~6.9° and attributed to the (100) plane of β-form of CoPc, was identified. This diffraction peak, specific to the β-form of CoPc, was also reported for organic films deposited by vacuum evaporation [28,30]. Regarding the inorganic thin film, although, in the powder form, ZnO shows a high crystallinity degree (Figure 1A), as a MAPLE-deposited thin layer, ZnO exhibits an amorphous nature, most probably its thickness (70 nm) being responsible for this behavior. A similar result was obtained when this metal oxide was deposited as a thin film at room temperature, without annealing treatments [31].

**Figure 4:**
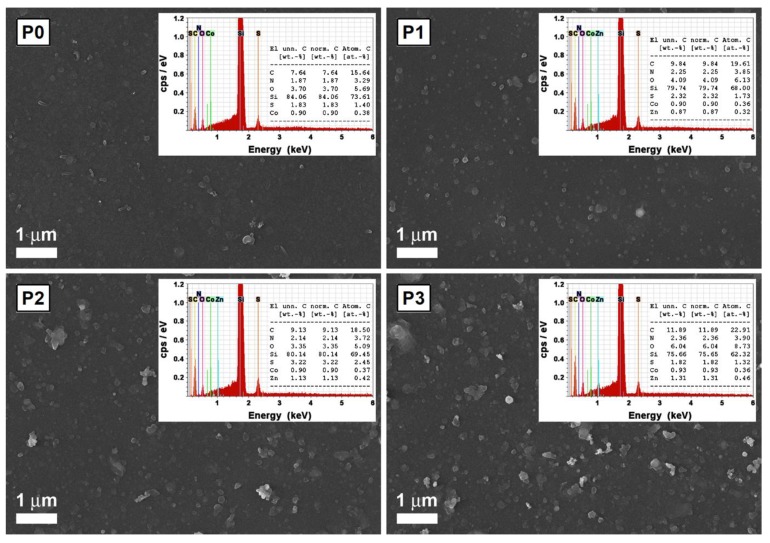
FESEM images of the MAPLE-deposited thin films. Insets: energy-dispersive X-ray analysis (EDX) spectra and weight and atomic percentages of the elements in the corresponding samples.

**Figure 5:**
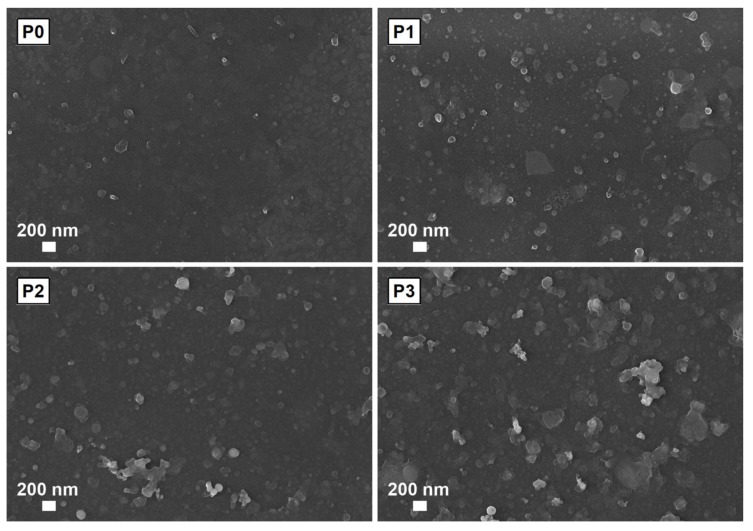
FESEM images at higher magnification of the MAPLE-deposited thin films.

**Figure 6:**
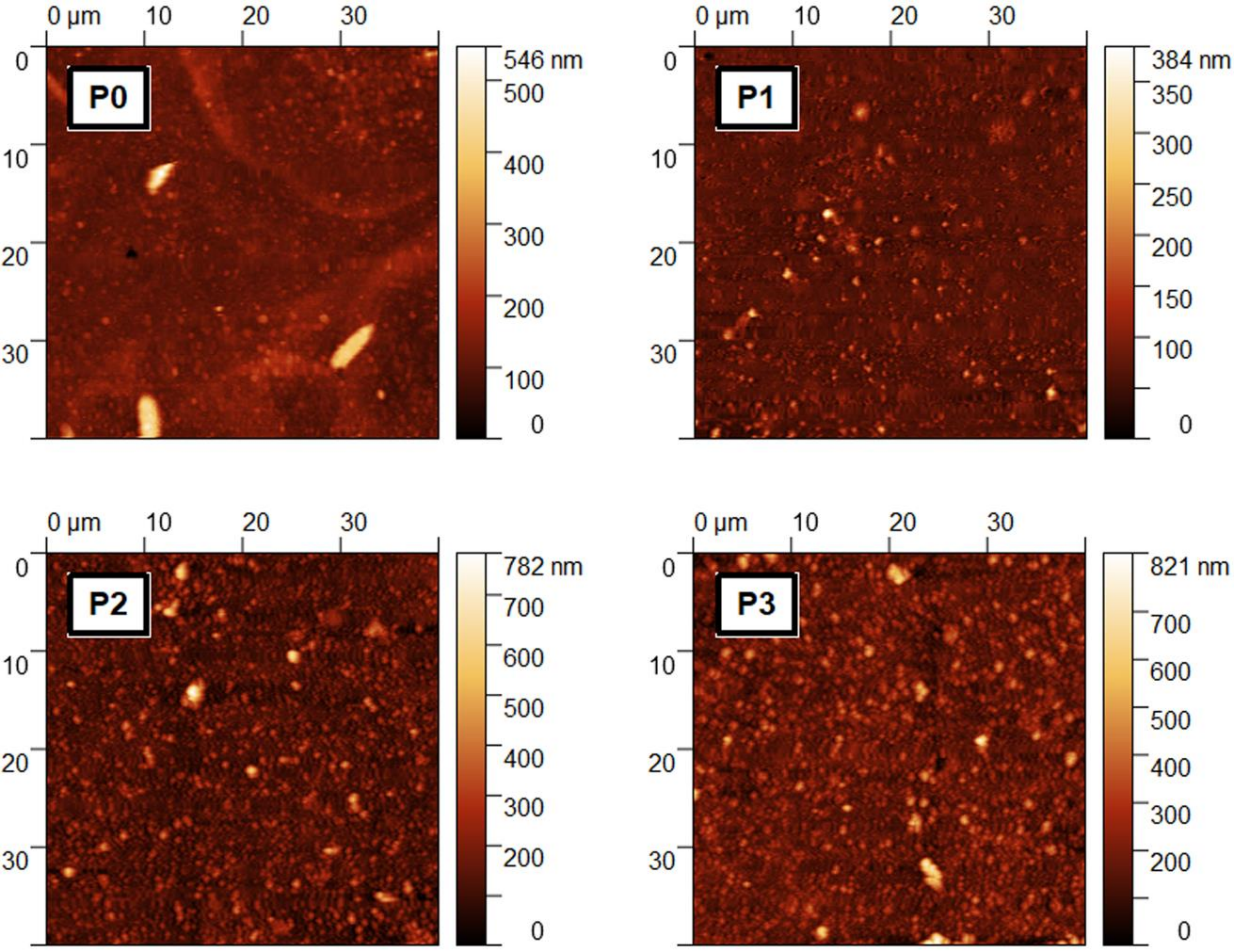
Topographic images of the MAPLE-deposited thin films.

MAPLE-deposited layers were investigated from morphological point of view, their FESEM images at two magnifications (Figure 4 and Figure 5) and AFM images (Figure 6) evidencing the globular morphology specific to films obtained by MAPLE process [19,20]. The elemental composition of these samples was also evaluated in order to confirm the presence of ZnO in the hybrid layers. Thus, in the EDX spectra of all investigated samples (Figure 4 insets), additionally to the signals corresponding to C, N and O elements, those attributed to S (from DMSO traces) and Si (from the deposition substrate) can be also observed. The simultaneous presence of Co and Zn peaks in the EDX spectra certify the coexistence of both CoPc and ZnO components in the hybrid nanocomposite layers. As expected, the Zn signal is missing in the P0 sample containing only the CoPc:C60 mixture, while in the P1–P3 samples containing CoPc:C60:ZnO hybrid nanocomposite, the Zn signal increases with the amount of the inorganic nanoparticles added into the organic mixture.

Also, the FESEM and AFM images of all investigated MAPLE layers disclose a clustering tendency, a similar effect being reported for blends containing two organic materials and metallic oxide nanoparticles [32]. The effect is more pronounced in the case of P1–P3 samples, as can be seen in the FESEM at higher magnification (Figure 5), the number of the aggregates enlarging with the ZnO quantity added in the MAPLE target used in the deposition of these hybrid nanocomposite layers.

Furthermore, the influence of the inorganic content added into the organic mixture on the surface and thickness of the MAPLE-deposited layers was explored. Thus, from the analysis of the topographic images (Figure 6), the values of the two roughness parameters, root mean square (RMS) and roughness average (Ra), were evaluated as being: 38 nm and 21 nm (P0 sample), 33 nm and 19 nm (P1 sample), 60 nm and 42 nm (P2 sample) and 67 nm and 48 nm (P3 sample). In comparison with the values of P0 sample, containing only CoPc:C60, those of P1 sample are almost similar suggesting that, in a small amount, the presence of ZnO nanoparticles does not affect the surface roughness. Hence, the higher values obtained in the case of P2 and P3 samples prove that the surface roughness of the hybrid nanocomposite layers is sensitive to the inorganic nanoparticles content.

Regarding the thickness of the MAPLE-deposited layers, the values estimated as being ~100 nm (P0 sample), ~200 nm (P1 sample), ~235 nm (P2 sample) and ~215 nm (P3 sample) indicate that the incorporation of the inorganic nanoparticles doubles the thickness of the hybrid nanocomposite layers. This result can be explained taking into consideration the size of about 20 nm of the ZnO nanoparticles (Figure 1E), their addition, even in a small quantity, resulting in a significant increase in the thickness value.

**Figure 7:**
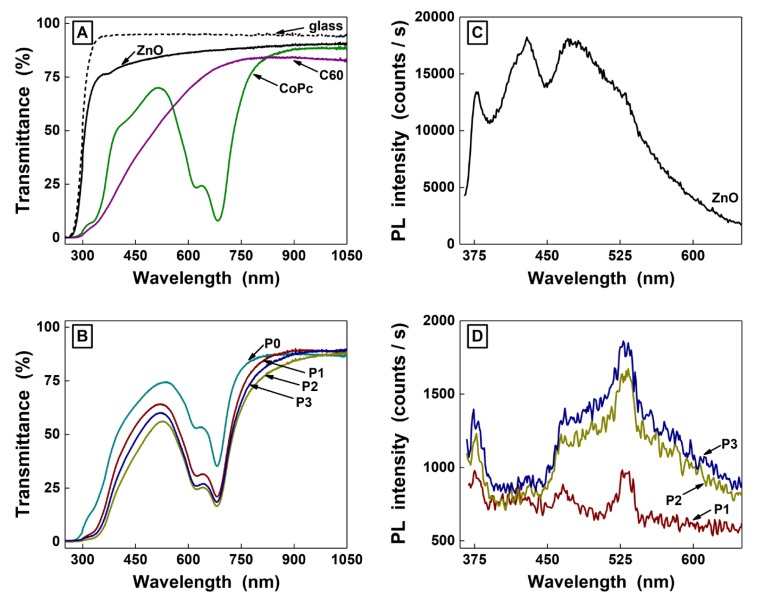
Ultraviolet–visible (UV–Vis) spectra (**A**,**B**) and PL spectra (**C**,**D**) of the MAPLE-deposited thin films.

The optical properties of the layers were assessed from the UV–Vis (Figure 7A,B) and PL spectra at λ_exc_ = 350 nm (Figure 7C,D). In order to obtain a proper evaluation of these features by avoiding the ITO influence, glass and silicon substrates were used in the UV–Vis and PL measurements, respectively.

The UV–Vis and PL spectra of the thin films based on the individual components deposited by MAPLE are presented in Figure 7A,C, respectively. Thus, the CoPc layer discloses the specific characteristics of phthalocyanines: the Soret band (also called B band) and the Q band presenting the Davydov split, with two maxima at around 625 nm (Q_1_) nm and 685 nm (Q_2_). The Q absorption bands are the result of π-π* (Q_1_) and excitonic (Q_2_) transitions in the phthalocyanine macrocyclic system having C and N atoms [33,34]. Additionally, the shape of the absorption spectrum with Q_2_ higher than Q_1_ is characteristic of the CoPc β-form [10]. The interactions between transition dipole moments of the neighboring molecules which have different orientations are responsible for the Davydov split [35]. In comparison with CoPc, the C60 layer reveals lower absorption in the visible range, its characteristic bands appearing in the UV domain [36].

In the case of the MAPLE layer based on ZnO nanoparticles, the UV–Vis spectrum shows a small shoulder at ~375 nm due to the ZnO band-to-band transition [11] whereas the PL spectrum discloses an UV emission band, peaked at ~375 nm, having an excitonic origin and a visible emission with more components, peaked at ~430 nm, ~480 nm and ~530 nm, originating from defect emissions [25,37]. The origin of these components has not yet been conclusively established, their mechanisms being still under debate [38]. The difference between the PL spectrum of the ZnO layer deposited by MAPLE and the PL spectrum of the chemically synthesized ZnO nanoparticles, which is dominated by the visible emission (Figure 1C), can be explained taking into account that the emission properties can be modified by the compound form, film or powder [37].

The UV–Vis and PL spectra of the MAPLE hybrid nanocomposite layers are given in Figure 7B,D, respectively. The UV–Vis spectrum of P0 sample is similar with that of CoPc film. If the UV–Vis spectra of the hybrid layers are dominated by the absorption characteristics of CoPc, the presence of ZnO inducing only a red shift of the fundamental absorption edge of CoPc, the PL spectra of the hybrid layers emphasize the emission features observed in the case of the ZnO layer. This result can be explained taking into account that the CoPc does not present photoluminescence due to strong spin orbit interaction [39] and the C60 in film form is usually featured by a weak emission [40]. Because the intensity of the emission bands is weaker in the hybrid layers in comparison with that of ZnO layer (the emission of ~430 nm being barely recognized), it can be assumed that a quenching effect appears when the ZnO nanoparticles are added to the CoPc:C60 mixture. However, the excitonic emission and the emissions of ~470 nm and ~530 nm (shoulder) can be still observed in the hybrid layers. It has to be mentioned that an artefact emission originating from the Xe flash lamp (observed also in reference [41]) is overlapped on the emission band that peaked at ~530 nm. Based on these data, it can be concluded that in the case of CoPc:C60:ZnO layers, the CoPc signature is dominant in the UV–Vis spectra while the ZnO signature prevails in the PL spectra.

**Figure 8:**
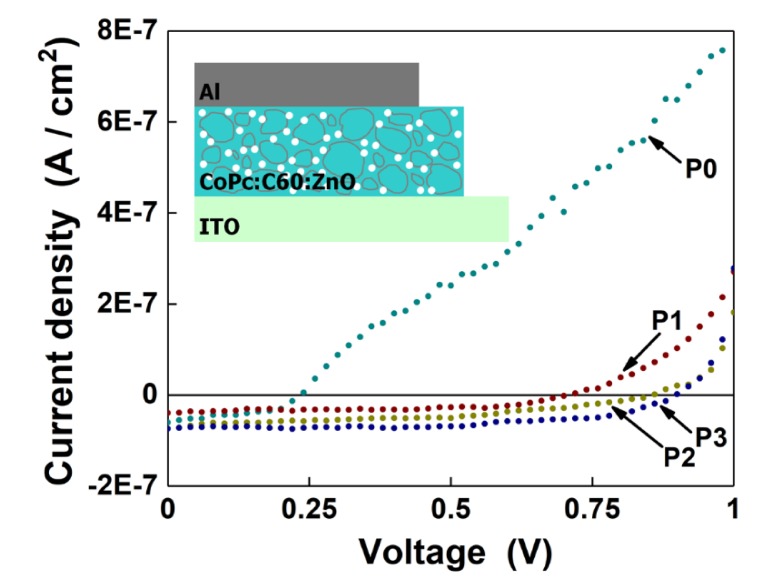
Current-voltage (J-V) characteristics of the structures based on the MAPLE-deposited layers. Inset: schematic representation of the structure developed with the MAPLE hybrid nanocomposite thin film.

The J-V characteristics of the hybrid nanocomposite layers, recorded under illumination, and a schematic representation of the investigated structures are given in Figure 8. The values of the electrical parameters, short-circuit current density (Jsc), open circuit voltage (V_oc_) and maximum power (P_max_), corresponding to each of the investigated structures are given in Table 1.

The structure based only on the p-n organic layer (P0 sample) is characterized by a smaller V_OC_ value compared to the structures developed with the hybrid nanocomposite layer (P1–P3 samples). This can be attributed to the presence of ZnO nanoparticles, similar V_OC_ values being also obtained for the hybrid structures embedding this metal oxide [42]. A recent study shows that the V_OC_ is dependent on the charge-carrier mobility, in both organic and hybrid solar cells, more specifically on the electron and hole mobility ratio [43]. It is known that the organic semiconductors have a low mobility comparatively with that of the inorganics. Considering the low hole mobility in CoPc (~4.42 × 10^−7^ cm^2^/Vs [30]), the presence of the high electron mobility (~6.6 × 10^−3^ cm^2^/Vs [44]) in ZnO leads to an increase in the V_OC_ value.

The J_SC_ value of P1 sample is lower in comparison with that of P0 sample, the P2 and P3 samples being characterized by higher values for this parameter. Most probably, the smaller quantity of the inorganic component added to the organic mixture is insufficient for promoting a larger number of dissociated excitons responsible for charge carriers arriving at the electrodes. By increasing the ZnO content, there is also an increase in the probability of dissociating more excitons, the metal oxide providing more electrons which can be collected at the electrodes [32]. Even if the values of the electrical parameters of these hybrid nanocomposite structures are small, further studies being necessary to improve them, the results prove that the addition of an appropriate amount of ZnO nanoparticles in the CoPc:C60 mixture results in an improvement of the J_SC_ value due to a more efficient charge transfer between the organic and inorganic components. Furthermore, the present study demonstrates that the MAPLE technique can be considered an alternative fabrication path in the deposition of hybrid photovoltaic structures.

## 4. Conclusions

Thin films based on CoPc:C60:ZnO hybrid nanocomposite were deposited by the MAPLE technique. ZnO powder was chemically synthesized by precipitation, a simple method which allows the preparation of metal oxide powder in large quantities involving inexpensive equipment and readily available raw materials. The synthesized ZnO powder contains nanoparticles with the size of about 20 nm characterized by structural and optical properties specific to this inorganic semiconductor. ZnO nanoparticles were embedded in the organic bulk heterojunction for fabricating thin films based on CoPc:C60:ZnO hybrid nanocomposite by the MAPLE technique. The FTIR spectra confirm that the chemical structure of both organic components, CoPc and C60, is preserved during the laser-deposition process. The XRD analysis shows that the MAPLE-deposited films are amorphous. The hybrid nanocomposite thin films present a globular morphology, typical to the layers deposited by MAPLE. Also, the FESEM and AFM images evidence a clusterization tendency which increases with the added ZnO amount. The EDX data confirm the coexistence of both CoPc and ZnO components in the hybrid nanocomposites layers. The optical investigations of the CoPC:C60:ZnO layers emphasize that the CoPc signature is dominant in their UV–Vis spectra while the ZnO signature prevails in their PL spectra. The electrical measurements carried out under illumination reveal that the structures containing the thin films deposited by MAPLE present a photovoltaic effect. Moreover, a certain amount of ZnO nanoparticles beside the CoPc:C60 in the hybrid nanocomposite layers improves their electrical parameters. This work proves the potential applications of hybrid nanocomposite layers in the photovoltaic cell’s area and the fact that MAPLE can be a viable alternative path in the fabrication of solar cell structures.

## Figures and Tables

**Table 1:** Electrical parameters of the MAPLE-deposited layers.

Sample	V_oc_ (V)	J_sc_ (A/cm^2^)	P_max_ (W)
P0	0.23	5.9 × 10^−8^	5.8 × 10^−9^
P1	0.71	4 × 10^−8^	1.5 × 10^−8^
P2	0.86	7.2 × 10^−8^	2.5 × 10^−8^
P3	0.9	7.5 × 10^−8^	3.6 × 10^−8^
